# New Bioactive Fused Triazolothiadiazoles as Bcl-2-Targeted Anticancer Agents

**DOI:** 10.3390/ijms222212272

**Published:** 2021-11-12

**Authors:** Rania Hamdy, Arwyn T. Jones, Mohamed El-Sadek, Alshaimaa M. Hamoda, Sarra B. Shakartalla, Zainab M. AL Shareef, Sameh S. M. Soliman, Andrew D. Westwell

**Affiliations:** 1 School of Pharmacy and Pharmaceutical Sciences, Cardiff University, Redwood Building, Cardiff CF10 3NB, UK; rania_hamdy2000@yahoo.com (R.H.); jonesat@cardiff.ac.uk (A.T.J.); 2 Faculty of Pharmacy, Zagazig University, Zagazig 44519, Egypt; m.elhussenyelsadek@yahoo.com; 3 Research Institute for Medical and Health Sciences, University of Sharjah, Sharjah P.O. Box 27272, United Arab Emirates; alshaimaahamoda@gmail.com (A.M.H.); u19106034@sharjah.ac.ae (S.B.S.); zalshareef@sharjah.ac.ae (Z.M.A.S.); 4 Department of Pharmacognosy, Faculty of Pharmacy, Assiut University, Assiut 71515, Egypt; 5 College of Medicine, University of Sharjah, Sharjah P.O. Box 27272, United Arab Emirates; 6 College of Pharmacy, University of Sharjah, Sharjah P.O. Box 27272, United Arab Emirates

**Keywords:** triazole, thiadiazole, indole, anticancer, Bcl-2 inhibitor, pharmacokinetics, synthesis, molecular modelling, apoptosis

## Abstract

A series of 3-(6-substituted phenyl-[1,2,4]-triazolo[3,4-b]-[1,3,4]-thiadiazol-3-yl)-1H-indoles (**5a**–**l**) were designed, synthesized and evaluated for anti-apoptotic Bcl-2-inhibitory activity. Synthesis of the target compounds was readily accomplished through a reaction of acyl hydrazide (**1**) with carbon disulfide in the presence of alcoholic potassium hydroxide to afford the corresponding intermediate potassium thiocarbamate salt (**2**), which underwent cyclization reaction in the presence of excess hydrazine hydrate to the corresponding triazole thiol (**3**). Further cyclisation reaction with substituted benzoyl chloride derivatives in the presence of phosphorous oxychloride afforded the final 6-phenyl-indol-3-yl [1,2,4]-triazolo[3,4-b]-[1,3,4]-thiadiazole compounds (**5a**–**l**). The novel series showed selective sub-micromolar IC_50_ growth-inhibitory activity against Bcl-2-expressing human cancer cell lines. The most potent 6-(2,4-dimethoxyphenyl) substituted analogue (**5k**) showed selective IC_50_ values of 0.31–0.7 µM against Bcl-2-expressing cell lines without inhibiting the Bcl-2-negative cell line (Jurkat). ELISA binding affinity assay (interruption of Bcl-2-Bim interaction) showed potent binding affinity for (**5k**) with an IC_50_ value of 0.32 µM. Moreover, it fulfils drug likeness criteria as a promising drug candidate.

## 1. Introduction

The anti-apoptotic Bcl-2 protein family influences oncogenesis signalling pathways through control of the intrinsic apoptotic process. The parent Bcl-2 protein is an apoptosis suppressor that is found to be frequently overexpressed in cancer cells [1], and the therapy-resistant cancer phenotype is commonly induced by the overexpression of anti-apoptotic Bcl-2. Therefore, targeting the anti-apoptotic Bcl-2 protein with small molecules that mimic the interaction of BH3 domain proteins with anti-apoptotic Bcl-2 can selectively induce apoptosis and is a promising strategy for the development of novel chemotherapeutic agents with potent anticancer efficacy [2].

**Figure 1:**
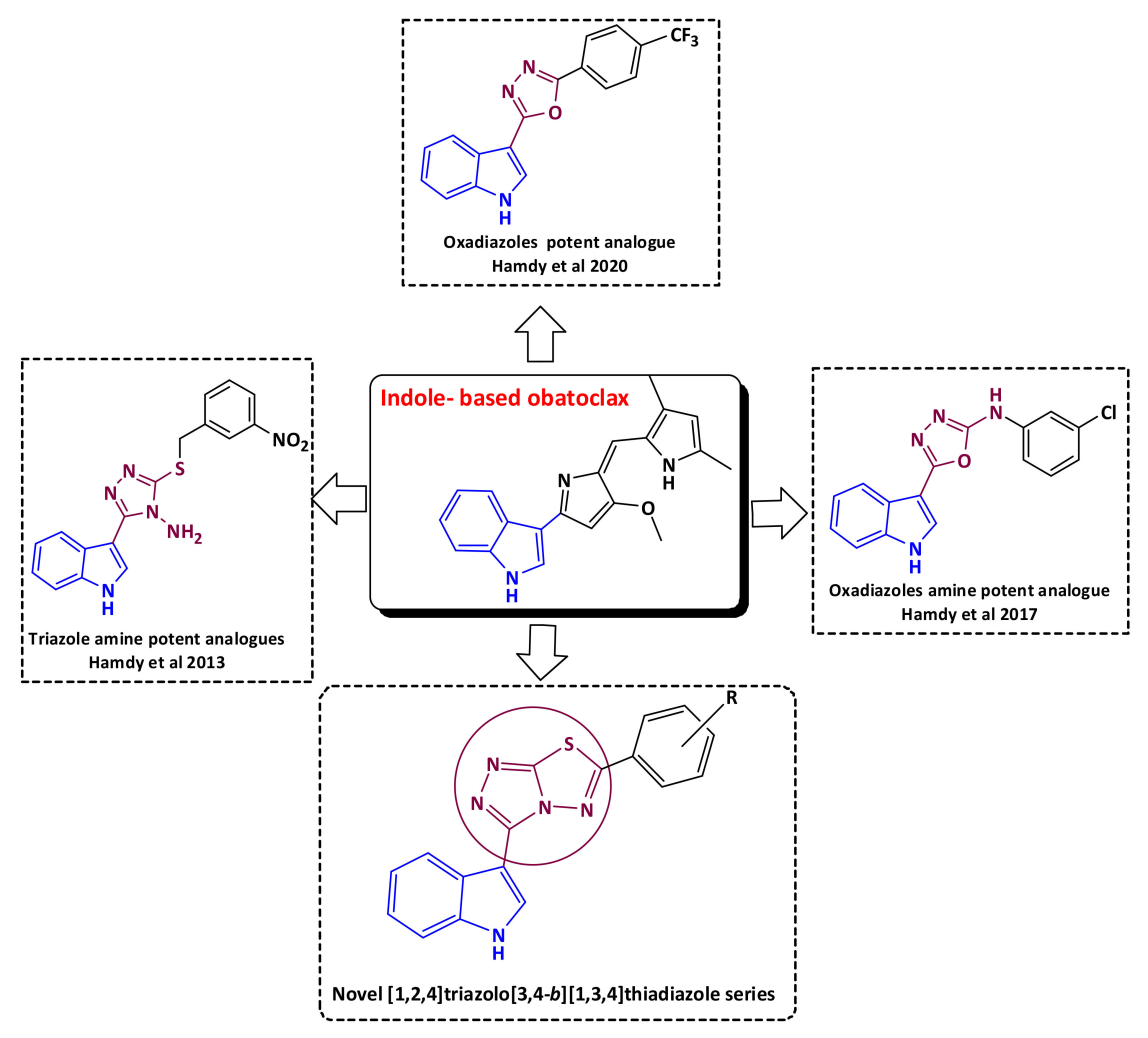
Rational design of the novel fused triazole-thiadiazole series.

**Scheme 1:**
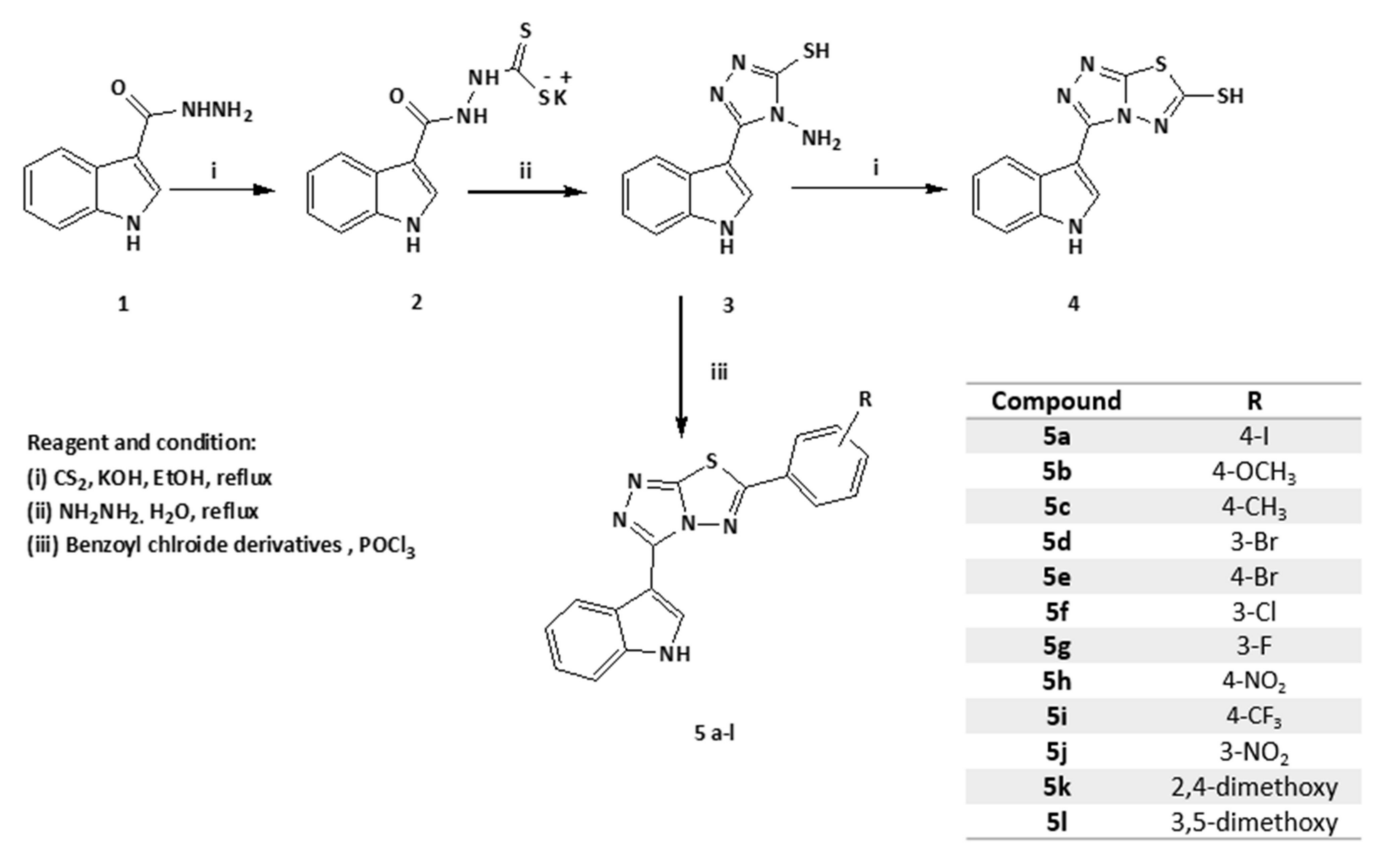
Synthesis of 3-(6-substituted phenyl-[1,2,4]-triazolo[3,4-b]-[1,3,4]-thiadiazol-3-yl)-1H-indoles (**5a**–**l**).

Indoles are nitrogen-based heterocyclic scaffolds that are found in most common FDA-approved anticancer drugs with the ability to sensitize cancer cells towards apoptosis [3]. Inspired by obatoclax as a clinically useful indole-based small molecule that inhibits the Bcl-2 protein [4], we previously designed and synthesized a series of indole-based compounds with oxadiazole [5,6,7] and triazole [8] scaffolds that show potent anticancer activity. Notably, the 1,3,4-thiadiazole scaffold was found to be an integral part of the anticancer agent moiety, with a toxophoric NCS moiety that may provide desirable pharmacophoric H-bond features alongside the lipophilic character of the sulphur atom [9]. Herein, we aimed to investigate the potential effect of incorporating a bioactive rigid [1,2,4]-triazolo[3,4-b]-[1,3,4]-thiadiazole scaffold within an indole-based structure with different substitutions (Scheme 1). These designed structures with enhanced physiochemical properties and fitting within the Bcl-2 binding site contribute to our continuous efforts to discover promising new potent therapeutics to combat cancer (Figure 1).

## 2. Results

### 2.1. Synthesis of the Entitled Compounds ***5a***–***l***

The reaction of 1H-indole-3-carboxylic acid hydrazide 1 with carbon disulphide and ethanolic KOH under reflux gave the corresponding potassium thiocarbamate salt 2 in 80% isolated yield, according to the previously reported method [10]. The intermediate thiocarbamate salt 2 was then reacted with hydrazine hydrate to provide the cyclized triazole thiol 3. Further cyclisation reaction with substituted benzoyl chloride derivatives in the presence of phosphorous oxychloride afforded the final 6-phenyl-indol-3-yl [1,2,4]triazolo[3,4-b][1,3,4]thiadiazole compounds (**5a**–**l**) Scheme 1. Cyclisation of intermediate 3 in the presence of carbon disulphide under basic conditions provided the thiazolo-thiazole-thiol 4.

### 2.2. Compound ***5k*** and ***5l*** Analogues Showed Potent and Selective Growth-Inhibitory Anticancer Activity on Bcl-2-Expressing Cell Lines

All the tested compounds (**4**, **5a**–**l**) were found to inhibit the growth of the Bcl-2-expressing human cancer cell lines (MDA-MB-231, HeLa and KG1a) with low micromolar IC_50_ values, except **5c**, which was inactive against the KG1A (Acute Myeloid Leukemia) cell line (Table 1). The Bcl-2-inhibitory natural product **gossypol** was used as a positive control. In general, test compounds showed weak activity against the Bcl-2-negative T-cell lymphocytic leukemia cell line (Jurkat) except compounds **5a** and **4**. Compounds (**5b**, **5c**, **5e**, **5i**, **5k** and **5l**) showed no activity against Jurkat cells, indicating that Bcl-2 could play a role in mediating the observed anticancer activity that later was confirmed through the ELISA binding assay. Considering the structure–activity relationships, the most active compound was **5k** (containing a 2,4-dimethoxyphenyl group), followed by **5l** (containing a 3,5-dimethoxyphenyl group). These observations reveal that the dimethoxyphenyl substitution was the most favourable for potent activity. In addition, the 4-methoxy **5b**, 4-trifluoromethyl **5i** and 4-bromo **5e** substitutions showed moderately potent inhibitory activity against Bcl-2-expressing cell lines. The different phenyl substitution patterns affected the physiochemical properties, spatial arrangement and orientation within the binding active site. A selection of the most potent and moderate active and selective compounds (**5b**, **5e**, **5i**, **5k**, **5l**) were further investigated for their competitive Bcl-2 binding affinity (ELISA assay).

### 2.3. ELISA Assay Showed Potent Competitive Binding Activity of ***5k*** and ***5l***

**Figure 2:**
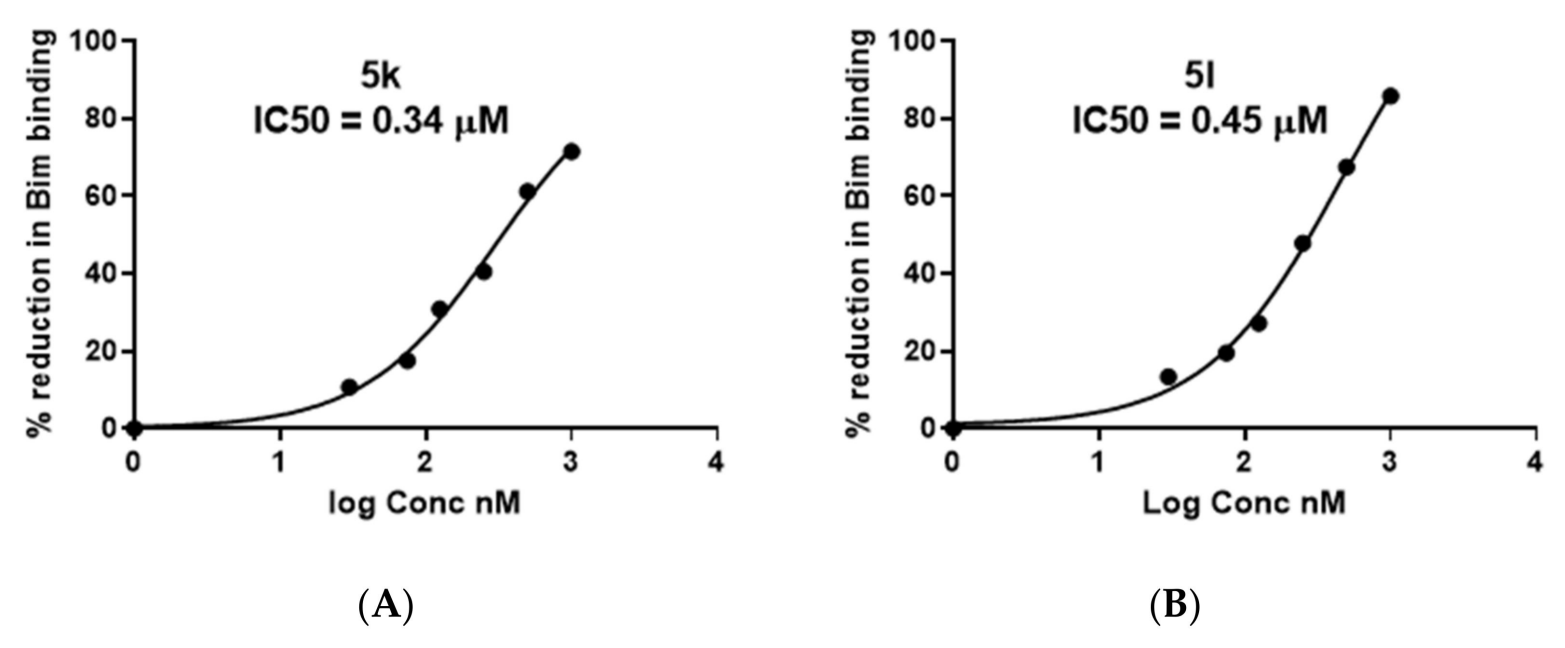
Inhibition activity of the most active compounds (**5k** and **5l**) against Bcl-2-Bim binding. (**A**) IC_50_ calculation of **5k**. (**B**) IC_50_ calculation of **5l**.

The most active compounds from the cell line assay (**5b**, **5e**, **5i**, **5k**, **5l**) were further evaluated according to their ability to compete with the pro-apoptotic BH3 domain Bim peptide for binding to His-tagged Bcl-2 protein, as previously described [6,11]. The results are shown in Figure 2 and Table 2. The natural product (−)-**gossypol** [12] was used as a positive control. Compounds (**5k** and **5l**) were found to be more potent than positive control **gossypol** in the binding assay (Table 2).

### 2.4. Molecular Docking to Rationalize the Potency and Selectivity of ***5k*** against Bcl-2

**Figure 3:**
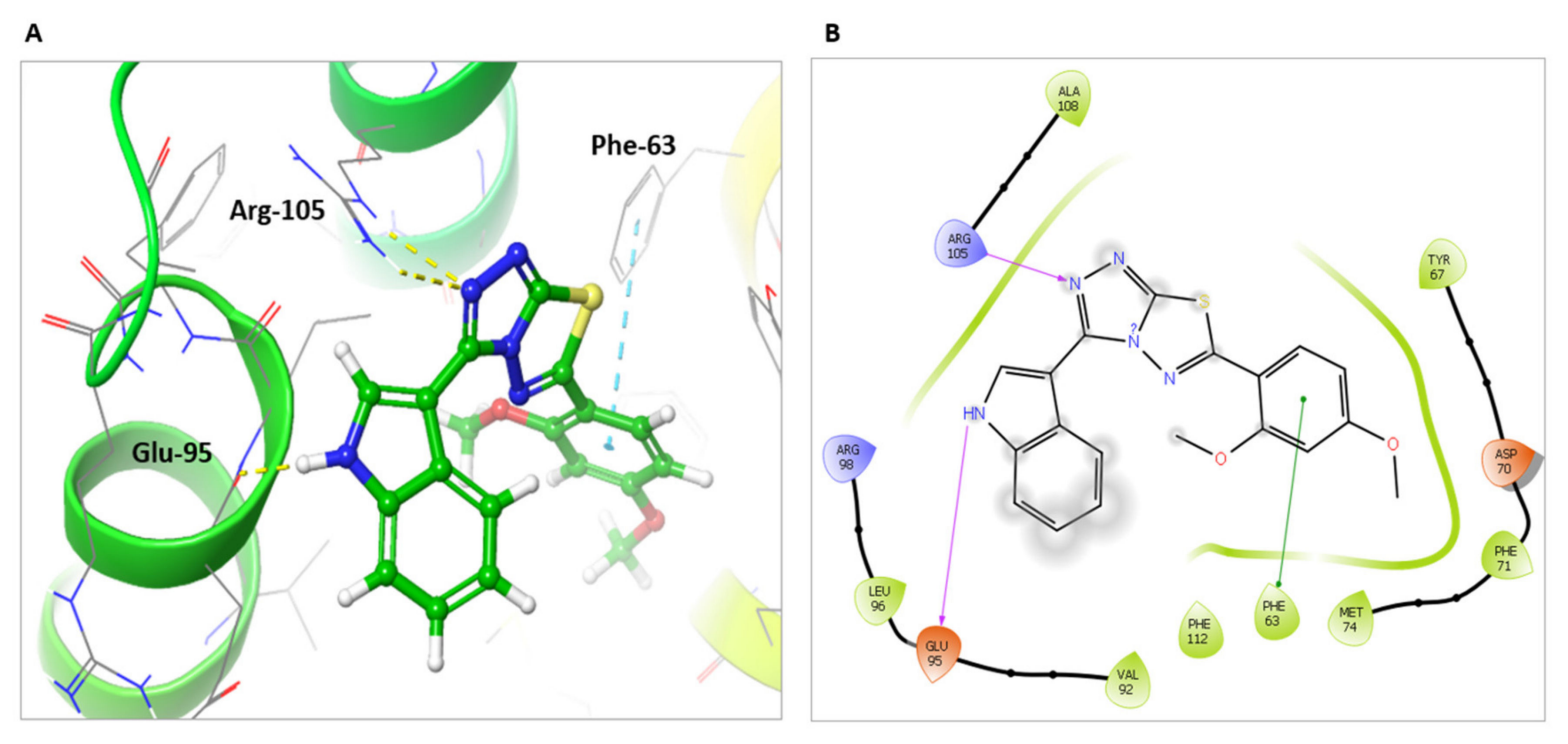
Molecular docking of **5k**. (**A**) Interaction of **5k** within the active site of Bcl-2 (PDB: 4AQ3) showed key interactions with Arg-105, Glu-95 and Phe-63. (**B**) Schematic diagram for ligand interaction of **5k**.

Compound **5k** showed stable interactions within the Bcl-2 binding site (4AQ3). The triazole nitrogen atom (N_2_) showed two H-bonds interacting with Arg-105; the indole nitrogen showed an H-bond interaction with Glu-95; and pi–pi stacking of the phenyl group with Phe-63 was also observed, along with hydrophobic interactions with Asp-70, Phe-63 and Try-67. These interactions provided an overall glide score of −5.8 kcal/mol (Figure 3).

### 2.5. ADME Predictions Confirm the Drug-Like Properties of Compounds

**Figure 4:**
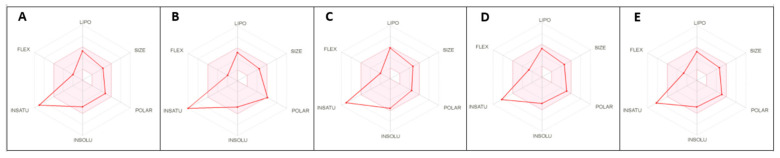
(**A**) Bioavailability radar of **5b**. (**B**) Bioavailability radar of **5e**. (**C**) Bioavailability radar of **5i**. (**D**) Bioavailability radar of **5k**. (**E**) Bioavailability radar of **5l**. The pink area indicates preferred properties range.

The new series and the most potent candidate complied with the Lipinski rule of 5, including hydrogen bond donors ((HBD) < 5, hydrogen bond acceptors (HBA) < 10, calculated octanol–water partition coefficient (cLog P) < 5 and molecular mass (Mwt) < 500) with predicted high GI absorption, moderate solubility, non-permeability of BBB and good bioavailability (Table 3). The compounds fulfil the criteria of drug likeness, indicating that they are promising drug candidates. The bioavailability radar pink area indicates the preferred properties range (Figure 4).

### 2.6. R-Group Analysis 

**Figure 5:**
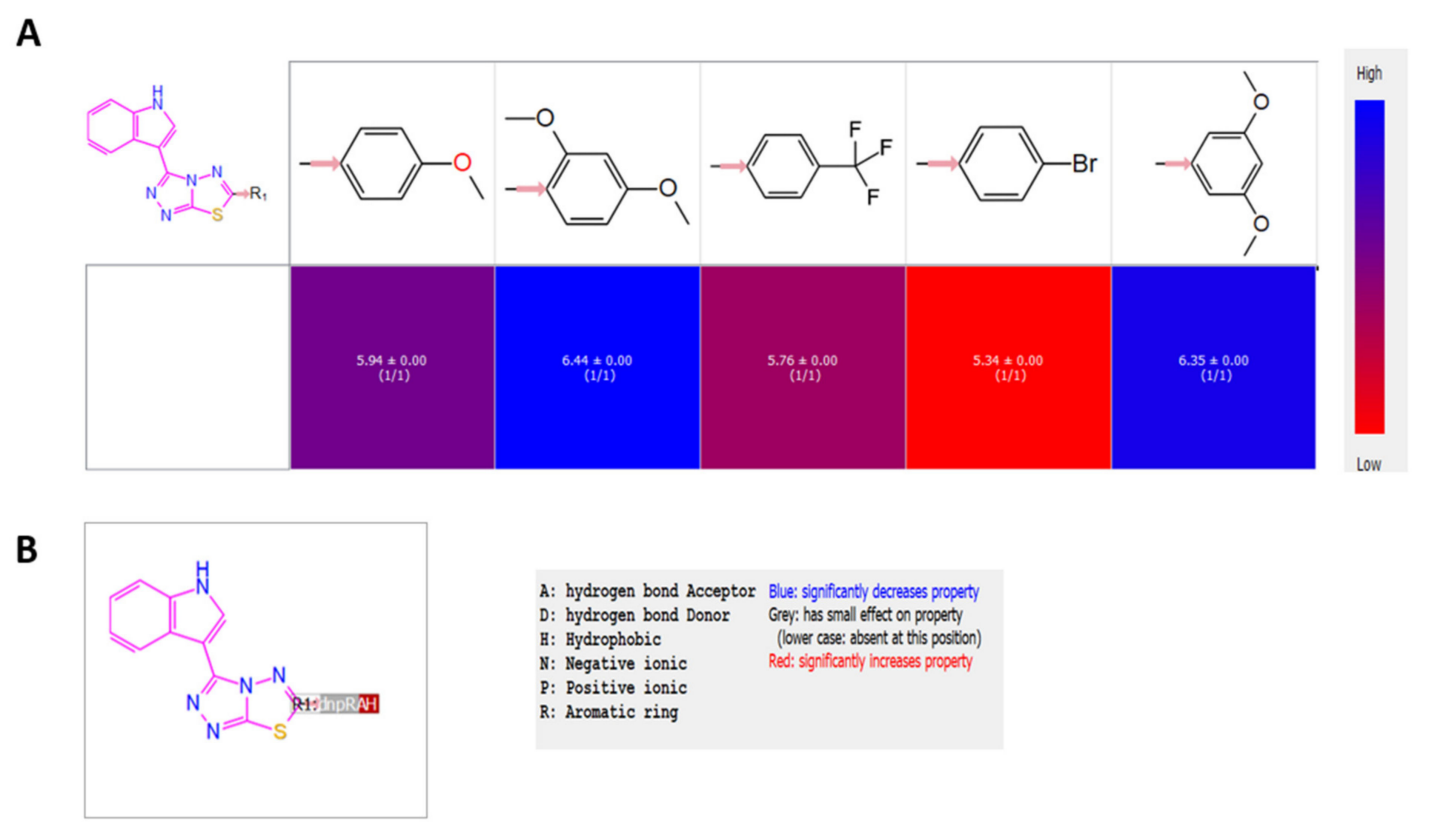
R-group analysis. (**A**) SAR analysis heat map. PIC_50_ values were displayed as colours ranging from red to blue as shown in the key. (**B**) QSAR analysis. Pharmacophoric features were displayed as colours between red, grey and blue, as shown in the key.

Structure-activity relationships (SAR) of the most potent and selective compounds (**5b**, **5e**, **5i**, **5k** and **5l**) were studied using the R-group analysis tool [13]. This analysis showed that the different structural substitution resulted in a variance in the biological activity and binding affinity. The different R-groups orientation showed dissimilar experimental binding activity with PIC_50_. The SAR heat map analysis showed different activities with different colours ranging from red to blue (Figure 5A). The superior activity was indicated with the 2,4-dimethoxyphenyl-substituted moiety of **5k**, followed by the 3,5-dimethoxyphenyl of **5l** that showed that the orientation of the dimethoxy substitution enhanced the activity. The 4-methoxyphenyl and the trifluoromethylphenyl substitution showed almost similar activity. The 4-bromophenyl substitution showed the least activity. Moreover, the pharmacophoric R-QSAR analysis highlighted that the hydrogen bond acceptor (HBA) and negative ionic interactions can significantly increase activity (Figure 5B). Therefore, the potent activity of **5k** and **5l** compounds is due to the dimethoxyphenyl substitution that has two oxygen-containing groups within the pharmacophoric HBA feature and negatively ionic feature, followed by one methoxy substitution. The trifluoromethylphenyl group of **5i** is negatively ionic with a weak HBA feature, while **5e** showed decreased activity with a weak HBA feature and less negatively ionic properties compared to the trifluoromethylphenyl substitution. Eventually, this information could be used for the future optimization of these compounds to significantly improve the anticancer activity.

## 3. Discussion

The indole-based Bcl-2 inhibitor obatoclax mesylate was used as the rational basis for the design and optimization of novel Bcl-2 inhibitors, presented in this study. Formerly, we reported the efficacy of the indole-based scaffold as potent and selective inhibitor of Bcl-2 protein [5,6,7,8]. The hybridization of indole-based structure with different heterocyclic moieties gained significant importance in the discovery of anticancer drugs. These hybrid structures showed unique physiochemical and pharmacokinetic properties. Further, hybridization of two pharmacophore scaffolds could reduce drug resistance and side effects [14]. The lipophilic property of the heterocyclic moiety enhanced its absorption and cellular uptake. The privileged heterocyclic moieties such as oxadiazole [14], triazole [15] and thiazole [11,16] possess a critical role in anticancer drug development. 

We previously prepared a series of indole-based oxadiazoles with different aromatic groups [5,6,7,8]. This led to the identification of a potent indole-based oxadiazole amine analogue with 3-chloro-phenyl substitution. The 3-chloro analogue showed a significantly potent IC_50_ value against Bcl-2-expressing human cancer cell lines, with a similar ELISA binding activity to **gossypol** as positive control [5]. Further structural optimization with different connecting groups aimed to improve the activity. SAR study revealed that direct connection of oxadiazole to the para trifluoromethyl phenyl substitution boosted the activity by two-fold more than the positive control **gossypol** using a Bcl-2 ELISA binding affinity assay [6].

While the indole-based triazole series were found less active compared to the corresponding oxadiazoles [8], herein, we investigated the potential role of novel indole-based compounds with rigid fused triazolo-thiazole scaffolds and different 6-position aromatic substitutions on the activity. This analysis shows that the different structural substitution resulted in a variance in the biological activity and binding affinity. The screening led to the identification of (**5b**, **5e**, **5i**, **5k** and **5l**) compounds as novel, selective and potent candidates. They showed a potent activity against Bcl-2-expressing human cancer cell lines, with limited inhibitory activity against a non-Bcl-2-expressing cancer cell line.

A competitive Bcl-2 ELISA assay recognizes compound **5k** (3-[6-(2,4-dimethoxyphenyl)-[1,2,4]-triazolo[3,4-b]-[1,3,4]-thiadiazol-3-yl]-1H-indole) as a potent Bcl-2 inhibitor, with two-fold lower IC_50_ value compared to (−)-**gossypol** positive control. The analysis highlights that the hydrogen bond acceptor (HBA) and negative ionic interactions significantly enhanced the activity. Hence, the potent activity of **5k** and **5l** could be attributed to the presence of two oxygen pharmacophoric HBA groups and the negatively ionic properties, followed by one HBA of **5b**. The **5i** compound has a negatively ionic fluorine atom with a weak HBA feature, while **5e** compound showed decreased activity with a weak HBA feature and less negatively ionic properties.

The study presented here is focused on early design, synthesis and SAR of novel triazolothiadiazole-based heterocycles as Bcl2-inhibitory anticancer hit compounds. Whilst we provide an early indication that the most active new compounds bind to Bcl-2 (with greater potency than the positive control (−)-**gossypol**), we are planning to further expand our study to substantiate the wider anticancer potential and mechanistic features of these active compounds such as **5k** and **5l**. This further work will include screening within a wider panel of cancer cell lines, including normal cell line controls.

A future aim for study will be to examine the mode of cytostatic/cytotoxic activity of the novel inhibitors alongside their effects on cell cycle; apoptosis; autophagy, given previous reports of G_0_/G_1_ arrest; and apoptosis induction for **gossypol** and obatoclax. [17,18,19] 

Finally, we note that overexpression of Bcl-2 in Bcl-2-negative cell lines may induce sensitivity to our new inhibitors. Similarly, knockdown of Bcl-2 in Bcl-2-overexpressing cell lines may induce resistance to the inhibitors. These future experiments will help to further clarify the role of Bcl-2 in mediating anticancer effects, as has been the case for related clinical agents such as venetoclax [20,21,22,23].

## 4. Materials and Methods

All computational work was carried on the Maestro graphical user interface of Schrödinger Suite 12.7 available at (www.schrodinger.com). Assay kit was purchased from (Thermo Scientific, Rockford, IL, USA). All chemical and reagent was purchased from (Sigma-Aldrich, Gillingham, UK).

### 4.1. Chemistry

Chemicals and solvents employed in chemical synthesis were of analytical grade and, when necessary, were purified and dried by standard methods. Reactions were monitored by thin-layer chromatography (TLC) using pre-coated silica gel plates (Kieselgel 60 F254, BDH), and spots were visualized under UV light (254 nm). Melting points were determined using a Gallenkamp melting point apparatus and are uncorrected. Column chromatography was performed with Merck silica gel 60 (40–60 μM). Both 1H NMR and 13C NMR spectra were recorded on a Bruker Avance 500 MHz spectrometer. Chemical shifts were expressed in parts per million (ppm) relative to tetramethylsilane. Coupling constant (J) values were represented in hertz (Hz), and the signals were designated as follows: s, singlet; d, doublet; t, triplet; m, multiplet. Mass spectroscopic data were obtained through electrospray ionization (ESI) mass spectrum (Bruker MicroTOF instrument). Elemental analysis (% CHN) was run by combustion analysis through an outsourced service (Medac Ltd., Surrey, UK).

#### 4.1.1. Synthesis of 4-Amino-5-(1H-indol-3-yl)-4H-[1,2,4]-triazole-3-thiol (**3**)

Carbon disulphide (12 mL, 20 mmol) was added dropwise to a cooled solution of indole-3-carboxylic acid hydrazide (1; 1.75 g, 10 mmol) and potassium hydroxide (0.85 g, 15 mmol) in ethanol (20 mL). The reaction mixture was stirred at room temperature for 16 h. After addition of dry diethyl ether (50 mL), the obtained product was treated without further purification with hydrazine hydrate (2 mL, 20 mmol) and water (4 mL) and heated at reflux for 1 h. The reaction was monitored by TLC, and after completion, water (10 mL) was added and neutralized with concentrated hydrochloric acid. The obtained solid was filtered, washed with cold water and recrystallized from ethanol. Yield 60%, mp: 160–162 °C. 1H-NMR (DMSO-*d*_6_): δ 6.49 (s, 2H, NH_2_), 7.19–7.25 (m, 2H, ArH), 7.45 (d, 1H, *J* = 7.5, ArH), 8.16 (d, 1H, *J* = 8.0, ArH), 8.45 (d, 1H, *J* = 2.25, ArH), 11.75 (s, 1H, NH of indole), 13.75 (s, 1H, SH).

#### 4.1.2. Synthesis of 3-(1H-Indol-3-yl)-[1,2,4]-triazolo[3,4-b]-[1,3,4]-thiadiazole-6-thiol (**4**)

In absolute ethanol (25 mL), 4-Amino-5-(1H-indol-3-yl)-4H-[1,2,4]-triazole-3-thiol (3, 2.5 g, 10 mmol) was dissolved in a solution of potassium hydroxide (0.6 g, 10 mmol). Carbon disulphide (15 mL) was added dropwise and heated at reflux for 18 h. The reaction mixture was evaporated under reduced pressure, the residue was purified by dissolving in 10% potassium hydroxide solution, and the cold filtrate was acidified with hydrochloric acid. The obtained solid was filtered, washed with water and recrystallized from aqueous ethanol. Yield 70%, mp: 170–172. 1H-NMR (DMSO-*d*_6_): δ 7.65–7.73 (m, 2H, ArH), 8.05 (d, 1H, *J* = 8.5, ArH), 8.65 (d, 1H, *J* = 7.9, ArH), 8.90 (d, 1H, *J* = 5.0, ArH), 12.50 (s, 1H, NH of indole), 13.95 (s, 1H, SH).

#### 4.1.3. General Preparation Procedure of 3-(6-Substituted phenyl-[1,2,4]-triazolo[3,4-b]-[1,3,4]-thiadiazol-3-yl)-1H-indol (**5a**–**l**)

Different benzoyl chloride derivatives (10 mmol) were added to a solution of the appropriate 4-amino-5-(1H-indol-3-yl)-4H-[1,2,4]-triazole-3-thiol (3, 2.5 g, 10 mmol) in phosphorous oxychloride (10 mL), and the mixture was heated at reflux for 8 h. The excess phosphorous oxychloride was removed under reduced pressure, ice water was added, and the precipitate was filtered and washed with aqueous NaHCO_3_ solution. The obtained product was recrystallized from ethanol.

3-[6-(4-Iodophenyl)-[1,2,4]-triazolo[3,4-b]-[1,3,4]-thiadiazol-3-yl]-1H-indole (**5a**). Yield 64%, mp: 183–185 °C. 1H-NMR (DMSO-*d*_6_): δ 7.21–7.31 (m, 2H, ArH), 7.57 (d, 1H, *J* = 5.4, ArH), 7.90 (d, 2H, *J* = 6.8, ArH), 8.04 (d, 2H, *J* = 6.8, ArH), 8.33 (d, 1H, *J* = 5.4, ArH), 8.42 (s, 1H, ArH), 11.85 (s, 1H, NH). 13C-NMR (DMSO-*d*_6_): δ 100.49 (C-3), 100.85 (ArCH), 112.10 (ArCH), 120.65 (ArCH), 121.00 (ArCH), 122.63 (ArCH), 124.25 (ArCH), 126.09 (ArCH), 128.69 (ArC), 128.81 (ArC), 136.25 (ArC), 138.42 (ArC), 143.82 (ArC), 151.54 (ArC), 165.27 (ArC). Calcd mass for C_17_H_10_IN_5_S: 442.87, found (*m*/*z*, ES+): 443.27.

3-[6-(4-Methoxyphenyl)-[1,2,4]-triazolo[3,4-b]-[1,3,4]-thiadiazol-3-yl]-1H-indole (**5b**). Yield 73%, mp: 192–194 °C. 1H-NMR (DMSO-*d*_6_): δ 4.30 (s, 3H, OCH_3_), 7.54–7.77 (m, 4H, ArH), 7.98 (d, 1H, *J* = 7.5, ArH), 8.46 (d, 2H, *J* = 9.5, ArH), 8.75 (d, 1H, *J* = 8.3, ArH), 8.81 (s, 1H, ArH), 12.24 (s, 1H, NH). 13C-NMR (DMSO-*d*_6_): δ 55.66 (OCH_3_), 100.95 (C-3), 112.09 (ArCH), 115.01 (ArCH), 120.62 (ArCH), 121.00 (ArCH), 121.49 (ArCH), 122.61 (ArCH), 124.24 (ArCH), 126.01 (ArC), 129.07 (ArC), 136.23 (ArC), 143.69 (ArC), 150.91 (ArC), 155.89 (ArC), 162.73 (ArC). Calcd mass for C_18_H_13_N_5_OS: 347.08, found (*m*/*z*, ES+): 348.40.

3-(6-p-Tolyl-[1,2,4]-triazolo[3,4-b]-[1,3,4]-thiadiazol-3-yl)-1H-indole (**5c**). Yield 75%, mp: 201–203 °C. 1H-NMR (DMSO-*d*_6_) δ 2.56 (s, 3H, CH_3_), 7.24–7.36 (m, 2H, ArH), 7.47 (d, 2H, *J* = 8.5, ArH), 7.57 (d, 1H, *J* = 7.8, ArH), 8.00 (d, 2H, *J* = 8.5, ArH), 8.33 (d, 1H, *J* = 7.1, ArH), 8.40 (d, 1H, *J* = 2.5, ArH), 11.82 (s, 1H, NH). 13C-NMR (DMSO-*d*_6_): δ 21.13 (CH_3_), 100.93 (C-3), 112.10 (ArCH), 115.21 (ArCH), 120.10 (ArCH), 120.63 (ArCH), 121.01 (ArCH), 122.62 (ArCH), 124.25 (ArC), 126.04 (ArC), 127.15 (ArC), 130.14 (ArC), 143.24 (ArC), 151.71 (ArC), 163.79 (ArC). Calcd mass for C_18_H_13_N_5_S: 331.09, found (*m*/*z*, ES+): 332.34.

3-[6-(3-Bromophenyl)-[1,2,4]-triazolo[3,4-b]-[1,3,4]-thiadiazol-3-yl]-1H-indole (**5d**). Yield 63%, mp: 179–182 °C. 1H-NMR (DMSO-*d*_6_): δ 7.21–7.30 (m, 2H, ArH), 7.55-7.65 (m, 2H, ArH), 7.91 (d, 1H, *J* = 8.0, ArH), 8.11 (d, 1H, *J* = 8.0, ArH), 8.33 (d, 2H, *J* = 6.6, ArH), 8.47 (s, 1H, ArH), 11.83 (s, 1H, NH). 13C-NMR (DMSO-*d*_6_): δ 100.74 (C-3), 112.14 (ArCH), 120.65 (ArCH), 120.65 (ArCH), 122.62 (ArCH), 122.72 (ArCH), 124.27 (ArCH), 126.36 (ArCH), 126.55 (ArCH), 129.34 (ArCH), 131.33 (ArC), 131.69 (ArC), 135.41 (ArC), 136.26 (ArC), 143.89 (ArC), 151.67 (ArC), 164.39 (ArC). Calcd mass for C_17_H_10_BrN_5_S: 394.98, found (*m*/*z*, ES+): 396.20.

3-[6-(4-Bromophenyl)-[1,2,4]-triazolo[3,4-b]-[1,3,4]-thiadiazol-3-yl]-1H-indole (**5e**). Yield 67%, mp: 196–198 °C. 1H-NMR (DMSO-*d*_6_): δ 7.19–7.29 (m, 2H, ArH), 7.6 (d, 1H, *J* = 7.5, ArH), 7.85 (d, 2H, *J* = 8.8, ArH), 8.09 (d, 2H, *J* = 7.5, ArH), 8.35 (d, 1H, *J* = 7.5, ArH), 8.49 (s, 1H, ArH), 11.94 (s, 1H, NH). 13C-NMR (DMSO-*d*_6_): δ 100.84 (C-3), 112.10 (ArCH), 120.64 (ArCH), 121.01 (ArCH), 122.62 (ArCH), 124.26 (ArCH), 126.11 (ArCH), 126.35 (ArCH), 128.43 (ArC), 129.11 (ArC), 132.60 (ArC), 136.25 (ArC), 143.83 (ArC), 151.07 (ArC), 164.93 (ArC). Calcd mass for C_17_H_10_BrN_5_S: 394.98, found (*m*/*z*, ES+): 396.60.

3-[6-(3-Chlorophenyl)-[1,2,4]-triazolo[3,4-b]-[1,3,4]-thiadiazol-3-yl]-1H-indole (**5f**). Yield 64%, mp: 185–187 °C. 1H-NMR (DMSO-*d*_6_): δ 7.29–7.35 (m, 2H, ArH), 7.59 (d, 1H, *J* = 7.3, ArH), 7.67 (m, 1H, ArH), 7.79 (d, 1H, *J* = 8.0, ArH), 8.07 (d, 1H, *J* = 6.3, ArH), 8.22 (d, 1H, *J* = 2.3, ArH), 8.36 (d, 1H, *J* = 7.0, ArH), 8.41 (s, 1H, ArH), 12.13 (s, 1H, NH). 13C-NMR (DMSO-*d*_6_): δ 100.77 (C-3), 112.11 (ArCH), 120.63 (ArCH), 121.00 (ArCH), 122.60 (ArCH), 124.27 (ArCH), 126.18 (ArCH), 126.33 (ArCH), 126.60 (ArCH), 131.15 (ArCH), 131.49 (ArC), 132.48 (ArC), 134.36 (ArC), 136.27 (ArC), 143.89 (ArC), 151.64 (ArC), 164.44 (ArC). Calcd mass for C_17_H_10_ClN_5_S: 351.03, found (*m*/*z*, ES+): 352.45.

3-[6-(3-Fluorophenyl)-[1,2,4]-triazolo[3,4-b]-[1,3,4]-thiadiazol-3-yl]-1H-indole (**5g**). Yield 74%, mp: 183–185 °C. 1H-NMR (DMSO-*d*_6_): δ 7.15–7.28 (m, 2H, ArCH), 7.54–7.58 (m, 2H, ArCH), 7.64–7.78 (m, 1H, ArCH), 7.93 (d, 1H, *J* = 8.3, ArH), 8.15 (d, 1H, *J* = 8.0, ArH), 8.47 (d, 1H, *J* = 6.8, ArH), 8.58 (s, 1H, ArCH), 11.87 (s, 1H, NH). 13C-NMR (DMSO-*d*_6_): δ 100.75 (C-3), 113.82 (ArCH), 114.01 (ArCH), 119.54 (ArCH), 119.71 (ArCH), 120.62 (ArCH), 121.00 (ArCH), 122.58 (ArCH), 123.67 (ArCH), 124.28 (ArCH), 126.34 (ArC), 131.81 (ArC), 136.29 (ArC), 143.88 (ArC), 151.59 (ArC), 161.40 (ArC), 163.36 (d, J-CF = 123.75). Calcd mass for C_17_H_10_FN_5_S: 335.06, found (*m*/*z*, ES+):336.36.

3-[6-(4-Nitrophenyl)-[1,2,4]-triazolo[3,4-b]-[1,3,4]-thiadiazol-3-yl]-1H-indole (**5h**). Yield 67%, mp: 196–198 °C. 1H-NMR (DMSO-*d*_6_): δ 7.22–7.32 (m, 2H, ArH), 7.57 (d, 1H, *J* = 7.9, ArH), 8.31 (d, 1H, *J* = 7.9, ArH), 8.37–8.47 (m, 5H, ArH), 11.94 (s, 1H, NH). 13C-NMR (DMSO-*d*_6_): δ 100.47 (C-3), 112.17 (ArCH), 117.29 (ArCH), 120.80 (ArCH), 121.00 (ArCH), 122.67 (ArCH), 124.20 (ArCH), 125.56 (ArCH), 126.33 (ArC), 127.07 (ArC), 131.12 (ArC), 140.43 (ArC), 143.18 (ArC), 156.26 (ArC), 163.34 (ArC). Calcd mass for C_17_H_10_N_6_O_2_S: 362.06, found (*m*/*z*, ES+): 363.03.

3-[6-(4-Trifluoromethylphenyl)-[1,2,4]-triazolo[3,4-b]-[1,3,4]-thiadiazol-3-yl]-1H-indole (**5i**). Yield 60%, mp: 194–196 °C. 1H-NMR (DMSO-*d*_6_): δ 7.22–7.31 (m, 2H, ArCH), 7.58 (d, 1H, *J* = 8.0, ArCH), 8.03 (d, 2H, *J* = 8.3, ArH), 8.27–8.33 (m, 3H, ArCH), 8.44 (s, 1H, ArCH), 11.88 (s, 1H, NH). 13C-NMR (DMSO-*d*_6_): δ 100.60 (C-3), 112.82 (ArCH), 117.01 (ArCH), 120.12 (ArCH), 121.00 (ArCH), 122.28 (ArCH), 125.08 (ArCH), 127.04 (ArCH), 131.85 (q, J-CF = 212.50), 136.29 (ArC), 143.54 (ArC), 151.45 (ArC), 155.09 (ArC), 161.34 (ArC), 163.47 (ArC). Calcd mass for C_17_H_10_F_3_N_5_S: 385.06, found (*m*/*z*, ES+): 386.45.

3-[6-(3-Nitrophenyl)-[1,2,4]-triazolo[3,4-b]-[1,3,4]-thiadiazol-3-yl]-1H-indole (**5j**). Yield 67%, mp: 196–198 °C.1H-NMR (DMSO-*d*_6_): δ 7.22–7.31 (m, 2H, ArH), 7.58 (d, 1H, *J* = 7.5, ArH), 7.96 (t, 1H, *J* = 6.5, ArH), 8.34 (d, 1H, *J* = 7.5, ArH), 8.45 (d, 1H, *J* = 2.5, ArH), 8.55 (d, 2H, *J* = 10.5, ArH), 8.80 (s, 1H, ArH), 11.87 (s, 1H, NH). 13C-NMR (DMSO-*d*_6_): δ 99.47 (C-3), 112.37 (ArCH), 116.59 (ArCH), 120.19 (ArCH), 120.95 (ArCH), 122.77 (ArCH), 124.01 (ArCH), 125.46 (ArCH), 126.92 (ArCH), 136.39 (ArCH), 140.89 (ArC), 145.11 (ArC), 151.07 (ArC), 156.56 (ArC), 157.37 (ArC), 160.14 (ArC), 161.27 (ArC). Calcd mass for C_17_H_10_N_6_O_2_S: 362.06, found (*m*/*z*, ES+): 363.11.

3-[6-(2,4-Dimethoxyphenyl)-[1,2,4]-triazolo[3,4-b]-[1,3,4]-thiadiazol-3-yl]-1H-indole (**5k**). Yield 73%, mp: 178–180 °C. 1H-NMR (DMSO-*d*_6_): δ 3.90 (s, 3H, OCH_3_), 4.09 (s, 3H, OCH_3_), 6.83 (dd, 1H, *J* = 6.5, 6.25, ArH), 6.90 (d, 1H, *J* = 2.5, ArH), 7.20–7.31 (m, 2H, ArH), 7.56 (d, 1H, *J* = 8.2, ArH), 8.34-8.36 (m, 2H, ArH), 8.42 (s, 1H, ArH), 11.80 (s, 1H, NH). 13C-NMR (DMSO-*d*_6_): δ 55.86 (OCH_3_), 56.52 (OCH_3_), 98.62 (C-3), 101.21 (ArCH), 107.93 (ArCH), 110.04 (ArCH), 112.04 (ArCH), 120.48 (ArCH), 121.07 (ArCH), 122.51 (ArCH), 124.32 (ArCH), 125.77 (ArC), 129.45 (ArC), 136.23 (ArC), 142.99 (ArC), 152.80 (ArC), 158.79 (ArC), 161.42 (ArC), 164.13 (ArC). Calcd mass for C_19_H_15_N_5_O_2_S: 377.09, found (*m*/*z*, ES+): 377.42.

3-[6-(3,5-Dimethoxyphenyl)-[1,2,4]-triazolo[3,4-b]-[1,3,4]-thiadiazol-3-yl]-1H-indole (**5l**). Yield 68%, mp: 193–195 °C. 1H-NMR (DMSO-*d*_6_): δ 3.90 (s, 3H, OCH_3_), 4.19 (s, 3H, OCH_3_), 6.84 (t, 1H, *J* = 8.8, ArH), 7.19 (d, 2H, *J* = 2.2, ArH), 7.21–7.29 (m, 2H, ArH), 7.59 (d, 1H, *J* = 8.2, ArH), 8.33 (d, 1H, *J* = 7.7, ArH), 8.43 (s, 1H, ArH), 11.68 (s, 1H, N). 13C-NMR (DMSO-*d*_6_): δ 55.75 (OCH_3_), 100.84 (C-3), 104.20 (ArCH), 105.24 (ArCH), 112.10 (ArCH), 120.60 (ArCH), 121.02 (ArCH), 122.59 (ArCH), 124.27 (ArCH), 126.17 (ArC), 130.90 (ArC), 136.24 (ArC), 143.80 (ArC), 151.53 (ArC), 161.11 (ArC), 165.62 (ArC). Calcd mass for C_19_H_15_N_5_O_2_S: 377.09, found (*m*/*z*, ES+): 377.63.

### 4.2. Biology

#### 4.2.1. Cell Viability—By MTT Assay

Human breast cancer (MDA-MB-231) and cervical cancer (HeLa) cells, obtained from the ATCC (Manassas, VA, USA), were maintained as previously described [5,16]. Cytotoxic effects of the tested compounds were assessed using MTT assay [6,17]. Briefly, 4000 cells in 0.1 mL of medium (Life Technologies, Paisley, UK) were seeded and allowed to adhere for 24 h [18]. The cells were treated with different concentrations of the tested compounds (from 0.00001 to 100 µM) and incubated for 72 h. In total, 20 µL of (5 mg/mL) MTT reagent (3-[4,5-dimethylthiazol-2-yl]-2,5-diphenyl tetrazolium bromide) in PBS was added and incubated for 4 h. The developed purple colour following the addition of DMSO was measured using a Multiskan Go machine (spectrophotometer) at 570 nm absorbance [5,8,11]. Each concentration experiment was repeated independently 6 times to establish reproducibility. The percentage cell viability was calculated following the formula below, and IC_50_ values were obtained by using GraphPad Prism 5 software (San Diego, CA, USA).

Cell viability (%) = [(OD test − OD blank) ÷ (OD negative control − OD blank)] × 100(1)

#### 4.2.2. Cell Viability—CellTiter-Blue^®^ Assay 

Human acute myeloid leukemia KG1a and acute T-cell lymphocytic leukemia Jurkat cells were cultured and maintained as previously described [5,6,7,8]. For each experiment, 15,000 cells in 0.1 mL of medium were seeded into a solid-black fluorescence 96-well plate and incubated for 24 h. The cells were treated with different concentrations of the test compounds (from 0.00001 to 100 µM) over a 10-fold dilution series and incubated for 24 h. CellTiter-Blue^®^ solution was added, followed by reading of the fluorescence according to our previous protocol [5,6,7,8]. IC_50_ values were obtained by using GraphPad Prism 5 software (San Diego, CA, USA). For each concentration, six independent experiments were carried out.

#### 4.2.3. Enzyme-Linked Immunosorbent Assay (ELISA)

ELISA assay was performed to evaluate the ability of the compounds to compete with Bim protein and inhibit its interaction with Bcl-2 protein. [5,6,7,8]. The addition of His-tagged Bcl-2 protein to a biotinylated Bim-coated plate according to our previously published protocol was followed by the addition of anti-His antibody (Abcam, Cambridge, UK) containing horseradish peroxidase enzyme (Qiagen, Crawley, UK), o-phenylenediamine (Sigma-Aldrich, UK) and hydrogen peroxide. The optical density was determined using a plate reader at wavelength of 450 nm. The experiments were carried out on three separate occasions, including both negative and positive controls. A plot of log nM concentration for each inhibitor against the percentage reduction in the Bim peptide was created, using non-linear regression curve analysis (GraphPad Prism 5, San Diego, CA, USA) and a software used to generate IC_50_ for the Bcl-2 inhibition activity.

### 4.3. Computational Design

#### Protein Preparation and Molecular Docking

The 3D crystal structure of human Bcl-2 (PDB ID: 4AQ3) protein was downloaded from the protein data bank (https://www.rcsb.org/ obtained on September 2021). The proteins were prepared and refined using the Protein Preparation Wizard Maestro to ensure structural correctness [19]. Crystallographic water molecules beyond 5 Å were removed. All the missing hydrogen atoms were added at pH 7.3 for appropriate ionization, and the tautomerization state of amino acid residues and proper bond order were assigned. Next, the refining of protein structures was performed, and the water molecules with <3 hydrogen bonds to non-waters were deleted. Finally, the energy minimization was done using OPLS4 to relieve steric clashes [20]. The ligand 2D structure were converted to 3D structure using LigPrep (Schrödinger) [21]. Hydrogen atoms were added, and the salt ions were removed. The subsequent energy minimization of each structure was carried out using OPLS4 force field [20]. The ligand in the crystal structure of Bcl-2 protein was used for grid generation. A grid box was generated at the centroid of the active site for docking studies, and the active site was defined around the ligand crystal structure. Molecular docking was performed within the catalytic pocket site of the proteins using standard precision (SP) mode of Grid using Glide [22,23] without applying any constraints. The prepared ligands were docked against grid generated bcl-2 (PDB: 4AQ3) in SP flexible mode [24]. 

### 4.4. Pharmacokinetics and Drug Likeness Filter and Target Prediction 

ADME properties of target compounds were predicted using Swiss-ADME server [25], using compounds in SMILES format [26]. Two different filters, Lipinski’s rule of five and Ghose filter, were used to evaluate the drug-like properties.

### 4.5. R-Group Analysis

The R-group mapping analysis was performed in the Schrödinger suite. First, the input LigPrep structure with the associated IC_50_ value was converted to PIC_50_ values. The maximum common core was defined with Combi-Glide bond labelling and alignment of structure for fingerprint similarity of side-chain to minimize the number of attached R-groups. The heat map analysis displayed the effect of different functional group position with different colour ranges that reflect its pIC_50_ activity. A QSAR model was generated based on pharmacophoric features such as hydrogen bond donor (D), acceptor (A), hydrophobic group (H), negatively ionizable (N), positively ionizable (P) and aromatic ring (R).

## 5. Conclusions

This study rationalized the design, synthesis and evaluation of novel series of (6-substituted phenyl-[1,2,4]-triazolo-[3,4-b]-[1,3,4]-thiadiazol-3-yl)-1H-indoles (**5a**–**l**) as novel inhibitors of Bcl-2 protein. The various aromatic substitutions and spatial orientations of the (**5a**–**l**) compounds within the Bcl-2 binding pockets afforded variable activity, while the most potent compound with sub-micromolar IC50 values was (**5k**), containing a (2,4-dimethoxy-phenyl) substitution. Moreover, molecular docking studies showed stable interactions of the (**5k**) compound within the BH3 domain of Bcl-2. This was confirmed with ELISA assay, which displayed a potent inhibitory IC50 value of 0.3 µM, representing a two-fold greater potency than **gossypol** as positive control. The predicted ADME properties showed that the compounds fulfil the criteria of drug likeness and preferable properties range. This indicates that they are promising drug candidates for further development as a lead compound against a Bcl-2 anti-apoptotic target.

## Figures and Tables

**Table 1:** Growth-inhibitory activity (IC_50_, µM) values for compounds (**4**, **5a**–**l**) against human cancer cell lines MDA-MB-231, HeLa, KG1a and Jurkat.

Compound		IC_50_^1^			
	R	MDA-MB-231	HeLa	KG1A	Jurkat
**4**	SH	15 ± 0.60	18.20 ± 0.60	28.45 ±0.43	10.35 ± 0.14
**5a**	4-I	2.72 ±0.32	0.54 ± 0.02	1.66 ± 0.03	8.12 ± 0.16
**5b**	4-OCH_3_	1.70 ± 0.61	2.70 ± 0.90	3.14 ± 0.19	>100
**5c**	4-CH_3_	16.28 ± 0.19	12.50 ± 0.23	>100	>100
**5d**	3-Br	4.9 ± 0.75	6.48 ± 0.67	20.15 ± 0.46	45.5 ± 0.40
**5e**	4-Br	8.29 ± 0.9	9.8 ± 0.3	4.9 ± 0.73	>100
**5f**	3-Cl	12.5 ± 0.66	3.60 ± 0.52	10.23 ± 0.65	20.5 ± 0.52
**5g**	3-F	7.5 ± 0.98	1.99 ± 0.28	8.6 ± 0.22	65.08 ± 1.25
**5h**	4-NO_2_	3.16 ± 0.32	22.13 ± 0.45	34.5 ± 0.30	58.09 ± 1.30
**5i**	4-CF_3_	4.22 ± 0.38	2.7 ± 0.19	2.19 ± 0.72	>100
**5j**	3-NO_2_	3.6 ± 0.28	2.18 ± 0.73	6.82 ± 0.45	32.25 ± 1.2
**5k**	2,4-dimethoxy	0.7 ± 0.3	0.57 ± 0.08	0.31 ± 0.32	>100
**5l**	3,5-dimethoxy	0.35 ±0.29	1.42 ± 0.17	1.15 ± 0.27	>100
**Gossypol**		5.5 ± 0.35	4.43 ± 0.54	4.2 ± 0.35	18.1 ± 1.3

^1^ Results are expressed as triplicate testing mean values ± SEM.

**Table 2:** The IC_50_ of the selected compounds in ELISA as binding assay for Bcl-2.

Compound	IC_50_ µM *
**Gossypol**	0.6 ± 0.09
**5b**	1.14 ± 0.09
**5e**	4.6± 0.19
**5i**	1.74 ± 0.15
**5k**	0.36 ± 0.05
**5l**	0.45 ± 0.03

* Mean value and SEM score of three independent measurements.

**Table 3:** Pharmacokinetic properties of the most active compounds as Bcl-2 inhibitor anticancer agents.

Compound	Mwt	HBA	HBD	TPSA	Ilogp	ESOL Class	Ali Log S	GI Absorption	BBB Permeation	Lipinski Violation	Bioavailability
**5b**	347	4	1	96.34	2.79	Moderately soluble	−5.39	High	No	0	0.55
**5e**	396.3	3	1	87.11	2.83	Moderately soluble	−5.95	High	No	0	0.55
**5i**	386.45	5	1	132.9	1.96	Moderately soluble	−6.02	Low	No	0	0.55
**5k**	377.42	5	1	105.6	3.05	Moderately soluble	−5.56	High	No	0	0.55
**5l**	377.42	5	1	105.6	2.94	Moderately soluble	−5.56	High	No	0	0.55

## Data Availability

The authors confirm that the data supporting the finding are available within the article.
